# Outcome of patients with nonmetastatic muscle‐invasive bladder cancer not undergoing cystectomy after treatment with noncisplatin‐based chemotherapy and/or radiotherapy: a retrospective analysis

**DOI:** 10.1002/cam4.685

**Published:** 2016-03-22

**Authors:** Aristotle Bamias, Petros Tsantoulis, Thomas Zilli, Athanasios Papatsoris, Francesca Caparrotti, Christos Kyratsas, Kimon Tzannis, Kostas Stravodimos, Michael Chrisofos, Gregory J. Wirth, Andreas Skolarikos, Dionysios Mitropoulos, Constantinos A. Constantinides, Charalambos Deliveliotis, Christophe E. Iselin, Raymond Miralbell, Pierre‐Yves Dietrich, Meletios A. Dimopoulos

**Affiliations:** ^1^ Hellenic Genito‐Urinary Cancer GroupAthensGreece; ^2^ Department of Clinical TherapeuticsMedical SchoolAthens UniversityAthensGreece; ^3^ Department of Oncology and Centre de Recherche Clinique Dubois FerrariGeneva University HospitalGenevaSwitzerland; ^4^ Department of Radiation OncologyGeneva University HospitalGenevaSwitzerland; ^5^ 2^nd^ Department of UrologyUniversity of AthensAthensGreece; ^6^ 1^st^ Department of UrologyUniversity of AthensAthensGreece; ^7^ Department of Urology DepartmentGeneva University HospitalGenevaSwitzerland

**Keywords:** Bladder cancer, multimodality treatment, unfit for cisplatin

## Abstract

Transurethral resection of bladder tumor (TURBT), radiotherapy, chemotherapy, or combinations can be used in patients with muscle‐invasive bladder cancer (MIBC) not undergoing cystectomy. Nevertheless, unfitness for cystectomy is frequently associated with unfitness for other therapeutic modalities. We report the outcome of patients with MIBC who did not undergo cystectomy and did not receive cisplatin‐based chemotherapy. Selection criteria for the study were nonmetastatic MIBC, no cystectomy, no cisplatin‐based chemotherapy. Chemotherapy and/or radiotherapy should have been used aside from TURBT. Forty‐nine patients (median age 79), managed between April 2001 and January 2012, were included in this analysis. Median Charlson Comorbidity Index was 5, while 76% were unfit for cisplatin. Treatment included radiotherapy (*n* = 7), carboplatin‐based chemotherapy (*n* = 25), carboplatin‐based chemotherapy followed by radiotherapy (*n* = 10), and radiochemotherapy (*n* = 7). Five‐year event‐free rate was 26% (standard error [SE] = 7) for overall survival, 23% (SE = 7) for progression‐free survival, and 30 (SE = 8) for cancer‐specific survival (CSS). Patients who were treated with combination of radiotherapy and chemotherapy had significantly longer CSS compared to those treated with radiotherapy or chemotherapy only (5‐year CSS rate: 16% [SE 8] vs. 63% [SE 15], *P* = 0.053). Unfit‐for‐cystectomy patients frequently receive suboptimal nonsurgical treatment. Their outcome was poor. Combining chemotherapy with radiotherapy produced better outcomes and should be prospectively evaluated.

## Introduction

Bladder cancer is one of the most frequent cancers, especially in the elderly population. In Europe, ~70% of patients are over 65 years old and many of them are current or former smokers with significant tobacco‐related morbidities. As a consequence, the management of bladder cancer requires particular attention to the needs of the frail and elderly who are often unable to tolerate or refuse standard therapies.

The standard of care for muscle‐invasive bladder cancer (MIBC) is surgery, typically radical cystectomy with pelvic lymph node dissection, prostatectomy, and often hysterectomy. Cisplatin‐based neoadjuvant chemotherapy is also recommended, since it significantly prolongs survival when added to surgery 1. In clinical practice, many patients with MIBC are unfit for radical surgery or unwilling to undergo surgery. This is more relevant for elderly populations. In the United States 2, only 42% of patients between 75 and 79 years old and 29% of patients between 80 and 84 years old underwent radical surgery. Patients not undergoing cystectomy are usually treated with multimodality approaches. These therapies usually employ transurethral resection of bladder tumor (TURBT) and a combination of radiotherapy and systemic chemotherapy. Still there are limitations in the use of optimal systemic therapy or radiotherapy in this group of patients.

The most effective chemotherapy in advanced bladder cancer consists of cisplatin‐based combination chemotherapy, which has also been mostly used in localized bladder cancer 1, 3, 4.

Not all patients with bladder cancer are fit to receive cisplatin‐based chemotherapy. Cisplatin is nephrotoxic and ototoxic and adequate hydration is essential to avoid kidney damage. In addition, combination chemotherapy is associated with myelotoxicity, while patients with poor performance status (PS) do not benefit from cisplatin‐based chemotherapy 5. Unfitness for cisplatin is well established in advanced bladder cancer 6, but it is also relevant for patients with localized disease only. Indeed, around 40% of surgically treated patients with MIBC 7 were ineligible for cisplatin treatment in a retrospective review because of renal function impairment. Fitness for cisplatin in medically inoperable patients is even less frequent as highlighted by another analysis showing that the majority of patients who were not treated surgically did not receive any major oncological treatment 8. Unfitness for cisplatin may also compromise optimal radiochemotherapy, since there is evidence that carboplatin is inferior to cisplatin also in this setting 4. In addition, unfit‐for‐surgery patients may also have compromised tolerance to a curative course of radiation therapy because of changes in physiologic reserves and functional status, influencing physicians toward less aggressive and less effective radiation therapy 9. Hypofractionation has been used 10 in order to increase tolerability of radiotherapy, but its efficacy compared to conventional fractionation remains questionable.

The previous data suggest that a sizable (albeit still not accurately determined) proportion of patients who are unfit for surgery are also unfit for optimal chemotherapy and/or radiotherapy. This “generally” unfit population is systematically underrepresented in large‐scale trials and little evidence is available to guide their management. Most data are retrospective and include small series of patients, usually analyzed together with fit‐for‐any therapy patients. Combinations of TURBT with chemotherapy, radiotherapy, or both have all been used. Neoadjuvant carboplatin‐based chemotherapy has been used in unfit‐for‐cisplatin patients 11, but it is currently unclear whether this treatment provides a meaningful benefit specifically to patients not undergoing cystectomy. Several issues such as optimal chemotherapy and radiotherapy regimes and selection of patients likely to benefit remain unresolved. Given these limitations, most treatment strategies are based on the available, mostly real world, data and on local preferences.

In order to focus to this unfit population, we performed a retrospective evaluation of the outcome of 49 patients deemed unfit for surgery and also not treated with cisplatin‐based chemotherapy, managed in a Swiss and a Greek tertiary care centers.

## Methods

### Patient selection

Patients included in this retrospective analysis were selected from the databases of a Greek (Department of Clinical Therapeutics, University of Athens) and a Swiss (Geneva University Hospital) Oncology Center based on the following criteria: histologically confirmed nonmetastatic MIBC, patients considered unfit for radical cystectomy, and no cisplatin‐based chemotherapy. TURBT alone was not accepted, but chemotherapy and/or radiotherapy should have also been administered. The following information was retrieved from the medical records of patients fulfilling these criteria: baseline characteristics (including comorbidities), chemotherapy, radiotherapy, disease progression, and death from disease or other causes. Clinical staging was reported by the referring urologist. Unfitness for cisplatin was assessed according to the criteria of Galsky et al. 12. Toxicity was assessed using NCI CTC v.4 grading for chemotherapy 13 and RTOG criteria for radiotherapy 14. The age‐adjusted Charlson Comorbidity Index (CCI) was calculated according to a standardized online application (http://farmacologiaclinica.info/scales/Charlson_Comorbidity/).

### Treatment

Although the type of chemotherapy or radiotherapy administered was not a criterion for patients selection, the fact that the oncological care of all patients was provided by only two centers resulted in fairly homogenous chemotherapy or radiotherapy treatment. The combination of carboplatin and gemcitabine 15 was used in all patients who received full chemotherapy except for one case, where the combination of methotrexate, carboplatin, and vinblastine 16 was used. Prophylactic use of granulocyte colony‐stimulating factors was not applied. Gemcitabine at a dose of 200 mg/m^2^ once weekly was used in all cases of concurrent radiochemotherapy.

In most cases, the radiotherapy schedule consisted of irradiation of the whole bladder with a delivered median dose of 60 Gy (range, 40–60 Gy) using a four‐field box three‐dimensional conformal radiotherapy technique. The whole pelvis was irradiated with a dose ranging between 40 and 45 Gy (median, 40 Gy) in 1.8 or 2 Gy per fraction. According to a previously published protocol 17, five patients underwent an exclusive whole pelvic radiotherapy (WPRT) with 40 Gy in 2 Gy daily fractions over 4 weeks, with a concomitant boost to the whole bladder of 20 Gy (10 × 2 Gy) delivered as a second daily fraction for the last 2 weeks of the treatment period (total dose 60 Gy). A 6‐h interval between the two daily fractions was left during the bifractionated period.

### Statistical analysis

The SPSS software was used for statistical analysis (SPSS for Windows, version 15.0, SPSS Inc. Chicago, IL, USA). Overall survival (OS) was measured from the date of chemotherapy or radiotherapy initiation until death from any cause or last follow‐up. For cancer‐specific survival (CSS) patients who died from other causes were censored at the time of death. Progression‐free survival (PFS) was measured from the date of chemotherapy or radiotherapy initiation until objective tumor progression, cancer death, or last follow‐up. Categorical variables were correlated using the chi‐square test. Age was categorized by cutoffs at 70 and 75 years, while for the categorization of hemoglobin, the cut off was set at 10 g/dL, based on previous publications, which reported an independent prognostic significance of this factor in urothelial cancer 18, 19. Time‐to‐event distributions were estimated using Kaplan–Meier curves and survival functions were compared across different groups with the log‐rank test. For multivariate analyses, the Cox proportional hazards model was used to assess the relationship of survival with various clinical and laboratory variables. The backward selection procedure with removal criterion (*P* > 0.10) based on likelihood ratio test was performed to identify significant variables. Throughout analysis, a level of 5% was used to denote statistical significance.

## Results

### Demographics

Forty‐nine patients (Greece: 33, Switzerland: 16), treated between April 2001 and January 2012, were included in this analysis. Their characteristics and treatment administered are shown in Table 1. Histological type was transitional cell carcinoma except for one adenocarcinoma case. Six patients had a clinical T3 stage and one patient had a clinical T4a stage. All the remaining patients had a clinical T2 tumor stage. Comorbidities were reported in 40 (89%) of 45 patients (no relevant information in four cases). The commonest comorbidities were coronary artery disease (*n* = 11), diabetes (*n* = 10), and hypertension (*n* = 9) (Appendix). Median CCI was 5 (3–8). All patients had at least a CCI of 3.

**Table 1:** Baseline and treatment characteristics of patients included in the analysis

Characteristic	*N* (%)
Total	49 (100)
Greece	33 (67)
Switzerland	16 (33)
Age	
Median (range)	79 (53–87)
>70	38 (77)
>75	32 (65)
Gender	
M	40 (82)
F	9 (18)
PS (*n* = 42)	
0	12 (28)
1	20 (48)
2	10 (24)
Hb (*n* = 43)	
Median (g/dL) (range)	13.2 (8.3–16.2)
≤10 g/dL	4 (9%)
CrCl (*n* = 45)	
≥60 mL/min	17 (38)
<60 mL/min	28 (62)
Clinical stage (*n* = 47)	
T2	40 (85)
T3	6 (13)
T4a	1 (2)
Charlson Comorbidity Index (*n* = 44)	
3	8 (18)
4	1 (2)
5	17 (39)
6	9 (20)
7	6 (14)
8	3 (7)
Unfit for cisplatin (*n* = 45)	34 (76)
PS ≥2^a^	10 (24)
CrCl <60 mL/min^a^	28 (62)
Hearing loss^a^	1 (2)
Preexisting neuropathy^a^	1 (2)
Heart failure^a^	4 (9)
Treatment	
Chemotherapy	25 (51)
Radiotherapy	7 (14)
Chemotherapy–radiotherapy	10 (21)
Chemoradiotherapy	7 (14)

Total number of patients with available relevant data in parentheses. PS, performance status; Hb, hemoglobin.

a Multiple criteria may coexist.

Fitness for cisplatin was assessable in 45 patients, 11 (24%) patients were fit for cisplatin. Six of these patients received chemotherapy, while five received radiochemotherapy. Among unfit‐for‐cisplatin patients, renal function impairment was common, with 65% of patients having a calculated creatinine clearance less than 60 mL/min.

### Treatment

Patients were treated with the following therapies: 25 received chemotherapy alone, 7 radiotherapy alone, 10 chemotherapy and subsequently radiotherapy, while 7 received concurrent radiochemotherapy. No patient underwent salvage cystectomy.

The median number of chemotherapy cycles administered to patients who received full chemotherapy was 7 (2–12). The median number of weekly gemcitabine doses administered as part of the radiochemotherapy regime was 6.

Age, clinical stage, and CCI were not correlated with the treatment administered. Patients receiving radiotherapy alone had worse PS than the rest (PS 2: 75% vs. 18%, *P* = 0.036). unfit‐for‐cisplatin patients formed the majority in every treatment group aside from that of radiochemotherapy, where 5 (71%) of 7 patients were fit for cisplatin (*P* = 0.002 for the comparison among the four treatment groups). This difference was due to a significantly higher percentage of patients with CrCl ≥60 mL/min (83% vs. 31% for the other treatment groups, *P* = 0.023).

### Toxicity

Worst recorded toxicities for chemotherapy or radiotherapy are shown in Table 2. Neutropenia (27%), fatigue (19%), nausea and vomiting (17%), and renal function deterioration (17%) were the most frequently recorded toxicities for chemotherapy. No neutropenic infection was reported. Grade 3/4 toxicities were rare with the exception of Grade 3/4 vascular toxicity which was reported in 4 (11%) cases. These consisted of deep venous thrombosis (*n* = 3) and ischemic stroke (*n* = 1). Two of the patients who suffered deep venous thrombosis had a history of coronary artery disease. The last patient had preexisting carotid artery stenosis and suffered a lethal stroke during chemotherapy. His death was considered treatment related.

**Table 2:** Worst reported toxicities (percentages in brackets)

Toxicity/Grade	1	2	3	4
Chemotherapy (*n* = 35)				
Neutropenia	5 (14)	3 (8)	2 (5)	0 (0)
Thrombocytopenia	3 (8)	1 (3)	1 (3)	0 (0)
Liver	1 (3)	1 (3)	1 (3)	0 (0)
Allergy	1 (3)	0 (0)	0 (0)	0 (0)
Renal function	4 (11)	1 (3)	1 (3)	0 (0)
Diarrhea	1 (3)	0 (0)	0 (0)	0 (0)
Nausea–vomiting	5 (14)	0 (0)	1 (3)	0 (0)
Non‐neutropenic infection	0 (0)	3 (8)	0 (0)	0 (0)
Vascular	0 (0)	0 (0)	3 (8)	1 (3)
Fatigue	5 (14)	2 (5)	0 (0)	0 (0)
Constipation	1 (3)	1 (3)	0 (0)	0 (0)
Arthralgia	0 (0)	1 (3)	0 (0)	0 (0)
Fever	0 (0)	1 (3)	0 (0)	0 (0)
Radiotherapy acute (*n* = 15)				
Genitourinary	4 (27)	5 (33)	0 (0)	0 (0)
Gastrointestinal	8 (53)	1 (7)	0 (0)	0 (0)
Radiotherapy late (*n* = 10)				
Genitourinary	2 (20)	0 (0)	0 (0)	0 (0)

Radiotherapy toxicity was mild. No Grade 3 or 4 toxicities were reported. In addition, no significant differences between radiotherapy and radiochemotherapy were observed.

### PFS and OS

The median follow‐up for the whole population was 68 months. During follow‐up, 27 patients experienced a relapse outside the bladder and 29 died, 24 due to disease progression and 5 (17%) due to the following causes: cardiac ischemia, stroke, heart failure, dementia, and shock. The first two patients had received chemotherapy, the third patient received radiotherapy alone, while the remaining two patients had received radiochemotherapy. From the 27 patients who relapsed, information about further treatment was available for 19. Only seven of them were treated upon relapse. They all received carboplatin‐based chemotherapy.

**Figure 1:**
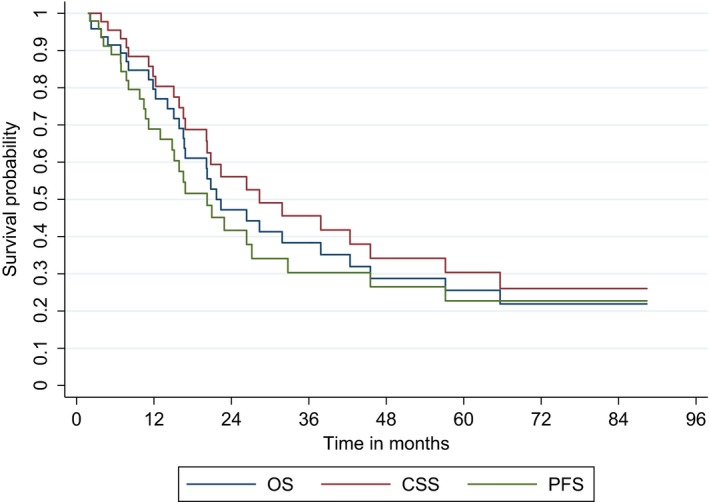
Overall survival (OS), progression‐free survival (PFS), and cancer‐specific survival (CSS) of 49 patients with muscle‐invasive bladder cancer who did not undergo cystectomy and were treated with noncisplatin‐based chemotherapy and/or radiotherapy.

**Figure 2:**
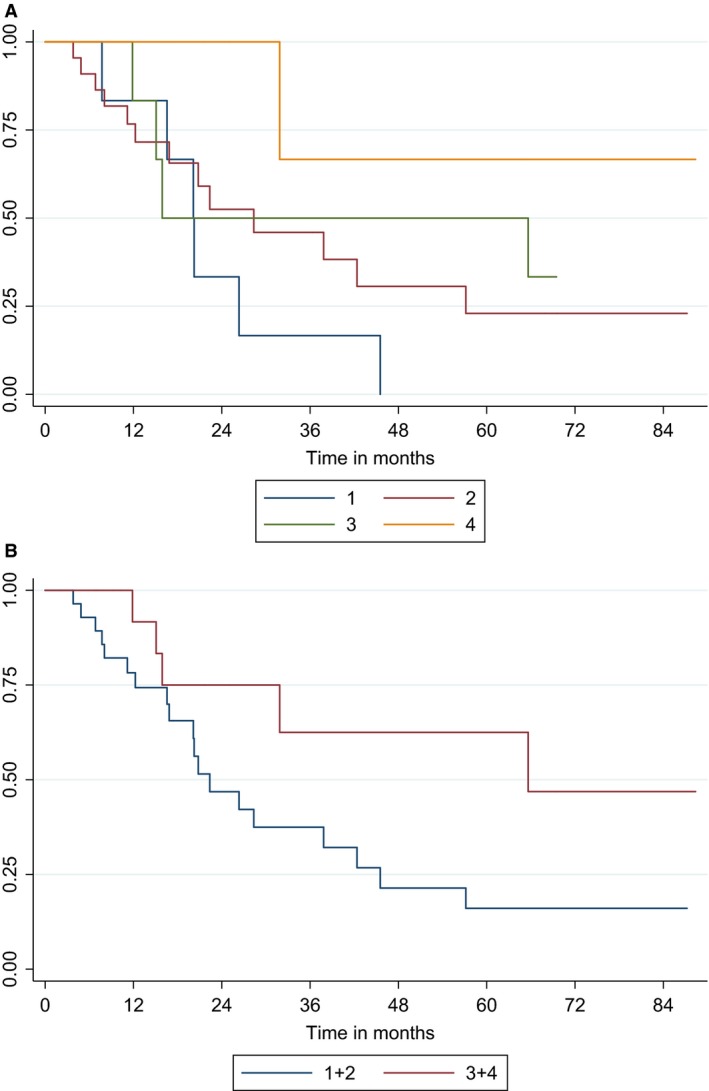
(A) Cancer‐specific survival (CSS) of 49 patients with muscle‐invasive bladder cancer who did not undergo cystectomy according to the treatment received. Radiotherapy alone (1), chemotherapy alone (2), chemotherapy followed by radiotherapy (3), and radiochemotherapy (4). (B) CSS of 49 patients with muscle‐invasive bladder cancer who did not undergo cystectomy according to the treatment received. Monotherapy (1 + 2) versus combination of chemotherapy and radiotherapy (3 + 4).

Median PFS, OS, CSS (Fig. 1), and the respective 5‐year event‐free rates are shown in Table 3. Significant differences according to treatment group were observed only in CSS analyses. The Kaplan–Meier curves for CSS according to treatment administered are shown in Figure 2A. Patients who received only radiotherapy had the worst CSS (*P* = 0.057 for the comparison radiotherapy vs. other treatment), while patients who received radiochemotherapy had the longest CSS (*P* = 0.054 for the comparison radiochemotherapy vs. other treatment). Radiotherapy alone was associated with inferior CSS compared with the combination of chemotherapy and radiotherapy (*P* = 0.021), while chemotherapy only was not (*P* = 0.123). When monotherapies and combinations were grouped, patients who received both radiotherapy and chemotherapy (sequential or concurrent) had better CSS compared to patients who received only one of these modalities (5‐year rate; standard error [SE]: 63% [15] vs. 16% [8], *P* = 0.053) (Fig. 2B). Eastern Co‐operative Oncology Group PS (ECOG PS) of 2 and CCI 6–8 were also associated with shorter OS, PFS, and CSS. Multivariate analysis of CSS showed that all three factors retained their prognostic significance (Table 4).

**Table 3:** Five‐year survival rate according to the treatment received

Overall (*n* = 49)	OS	PFS	CSS
	26 (7)	23 (7)	30 (8)
Treatment			
R (*n* = 7)	0	0	0
C (*n* = 25)	20 (10)	21 (10)	23 (11)
C–R (*n* = 10)	50 (20)	39 (20)	50 (20)
C+R (*n* = 7)	44 (22)	53 (24)	67 (27)
R/C (*n* = 32)	14 (7)	14 (7)	16 (8)
C–R/C+R (*n* = 17)	49 (14)	48 (15)	63 (15)
R/C+R (*n* = 14)	19 (11)	15 (13)	26 (15)
C/C–R (*n* = 35)	29 (9)	25 (9)	32 (10)

Standard errors in parentheses. OS, overall survival; PFS, progression‐free survival; CSS, cancer‐specific survival; R, radiotherapy; C, chemotherapy; C–R, chemotherapy followed by radiotherapy; C+R, concurrent radiochemotherapy.

**Table 4:** Uni‐ and multivariate analysis for cancer‐specific survival

Factor	5‐year SR% (SE)	Log‐rank *P*	Univariate		Multivariate	
			HR (SE)	*P*	HR (SE)	*P*
ECOG PS		0.013		0.037		0.018
0	88 (12)		1		1	
1 + 2	20 (9)		8.60 (8.87)		12.20 (12.90)	
CCI		0.013		0.017		0.023
3–5	44 (13)		1		1	
6–8	17 (11)		2.88 (1.28)		2.75 (1.23)	
Treatment group		0.053		0.062		0.038
R/C (*n* = 32)	16 (8)		1		1	
C–R/C+R (*n* = 17)	63 (16)		0.39 (0.20)		0.32 (0.18)	

ECOG, Eastern Co‐operative Oncology Group; PS, performance status; CCI, Charlson Comorbidity Index; SR, survival rate; SE, standard error; HR, hazard ratio; R, radiotherapy; C, chemotherapy; C–R, chemotherapy followed by radiotherapy; C+R, concurrent radiochemotherapy.

## Discussion

Patients with nonmetastatic MIBC, not undergoing cystectomy, represent a highly heterogeneous group. For this reason, their management remains a challenge. Traditionally, studies of such patients group together fit and unfit for surgery patients. It is, however, obvious that treatment options between these two groups differ considerably. Bladder preservation trimodality therapy has been traditionally used to manage patients with MIBC not undergoing cystectomy. Most such studies have used cisplatin‐based chemotherapy and radiochemotherapy reporting outcomes comparable to those of cystectomy series 3, 4, 20, 21 (Table 5). Nevertheless, 40% of patients with localized bladder cancer cannot receive cisplatin. In addition, it has been suggested that unfit‐for‐surgery patients may also be unfit for optimal radiotherapy 9. Finally, the excellent results reported with bladder preservation strategies are always associated with a cystectomy rate of 15–20% in cases of incomplete response to radiochemotherapy or muscle‐invasive relapses 3, 4, 22. This is not an option for unfit‐for‐surgery patients. Thus, unfitness for surgery may also infer (for a sizeable, albeit currently undetermined, percentage of patients) general “unfitness” for optimal management.

**Table 5:** Results of recent series of patients with nonmetastatic, muscle‐invasive bladder cancer not undergoing cystectomy

Author/reference	*n*	Treatment	5‐year OS rate	5‐year CSS rate
Rodel et al. 4	415 (89 T1 tumors)	R, R+Cis^a^, R+Ca^a^	R 40%R+Cis 62%^a^R+Ca 45%	All 45% (324 MIBC patients)
Efstathiou et al. 3	348	R+Cis^b^	52%	64%
Herr 24	63	Ccis	64%^c^	
Chung et al. 20	340 (36 T1 tumors)	R, R+Cis^a^, R‐Cis^d^	32%	42%
James et al. 22	360	R, R + 5‐FU/MMC^a^ (neoadjuvant Ccis 33%)	R 35%R + 5‐FU/MMC 48%	
Mitin et al. 21	93	R^bf^+cis+pac/5FU^b^	R+cis+pac 71%R+cis+5FU 75%	
Current study	49	R, Cca, R+Ca/G^e^	26%R NRCca 20%R+Ca/G 49%	All 30%R 23%Cca 40%R+Ca/G 63%^b^

Cis, cisplatin; Ca, carboplatin; G, gemcitabine; 5‐FU, 5‐fluorouracil; MMC, mitomycin C; pac, paclitaxel.

a Concurrent radiochemotherapy.

b Peri‐radiation chemotherapy and concurrent radiochemotherapy; Ccis: cisplatin‐based chemotherapy.

c Median follow‐up 86 months and minimum follow‐up 5 years; Cca: carboplatin‐based chemotherapy.

d Neoadjuvant chemotherapy followed by radiotherapy.

e Ten patients received chemotherapy (carboplatin based) followed by radiotherapy and seven patients received concurrent radiochemotherapy (gemcitabine a: *P* < 0.05 for comparisons with R and R+Ca; b: *P *<* *0.05 for comparison with R; bf: bifractionation.

For the above reasons, we specifically included patients who did not undergo cystectomy due to medical unfitness and who, at the same time, did not receive cisplatin‐based chemotherapy or radiochemotherapy using cisplatin. Although we did not set specific fitness criteria for surgery or cisplatin, it seems that we selected a generally “unfit” population: all patients had at least a CCI of 3, while 80% had a CCI of at least 5. Such CCI scores are associated with increased postsurgical mortality after urological procedures 23. In addition, 76% of our patients were truly unfit for cisplatin according to the contemporary criteria 12. We did not set specific criteria for unfitness for optimal radiotherapy. Nevertheless, 24% of our patients had a PS of 2. This is in contrast to the 3% in the study by James et al. 22, while other recent studies did not include any patients with a PS of 2 21. This together with the high CCI suggests that many of our patients could have been considered poor candidates for optimal radiotherapy. The fact that the fitter patients were selected for bifractionated radiochemotherapy supports an association of frailty with less intense radiotherapy. We, therefore, believe that this retrospective analysis offers useful real‐world information regarding both the characteristics as well as the outcome of unfit patients with nonmetastatic MIBC.

Our analysis has certain limitations. The number of patients included in this study is relatively small, while its retrospective nature as well as the inclusion of patients treated in two specializing centers may have introduced biases in the selection of patients as well as the quality of their management, which might have been superior to that of the general community practice. In addition, our patients were treated over a period of 10 years, during which the perception of surgical fitness and/or utilization of chemotherapy and radiotherapy may have changed. Unfitness for surgery was assessed by treating surgeons. Therefore, a degree of variability in this selection may exist. It should be noted, however, that our patients were treated in two tertiary referral centers and were evaluated by urologists specializing in urological cancer. In addition, the baseline characteristics of these patients suggest that referring surgeons mainly followed common practice for unfitness for surgery. Although the updated EAU guidelines do not include criteria for unfitness for surgery, they support the use of validated comorbidity scales, such as the age‐adjusted CCI to make such a decision. The radicality of TURBT could not be studied. The radicality of TURBT and the frequency of repeat cystoscopy may be compromised in frail and unfit patients not only due to their comorbidities, but also lack of motivation and this is likely to have been the case for our series.

Optimal nonsurgical treatment cannot be currently defined for the population studied here, since management is inevitably individualized according to the available therapeutic options. Indeed, four different therapies (radiotherapy, chemotherapy, sequential chemotherapy and radiotherapy, and radiochemotherapy) were applied in our series. Both chemotherapy and radiotherapy were homogenous and well tolerated. This is particularly important for radiotherapy, since the protocols used were not intended specifically for frail and unfit patients. The outcome of our patients was poor with only 26% surviving 5 years. This appears inferior to that of previous series of patients not undergoing cystectomy (Table 5). The major difference with our study is that the majority or all patients included in previous studies were fit for surgery. The reason for the poor outcome in our series is most likely multifactorial. The impact of possibly less aggressive TURBT, which is a crucial component in all trimodality protocols, has already been discussed. Comorbidities and advanced age may also represent another factor since 17% of deaths were due to the causes other than cancer. The same factors may also have influenced decisions against treatment at relapse, since chemotherapy at relapse was used in only 25% of cases. Chemotherapy was suboptimal due to the unfitness for cisplatin in the majority of cases. A 63% 5‐year survival was reported for a series of 63 fit for surgery patients who refused surgery and were managed with cisplatin‐based chemotherapy only 24. The respective percentage in our series was 20%. The use of carboplatin instead of cisplatin alone cannot explain this difference. Poorer PS at baseline, inadequate follow‐up of poorly motivated patients, and unfitness for surgery in the case of muscle‐invasive relapse may have also contributed to the poor outcome. Radiotherapy was also not optimally used. Patients with poor PS received only radiotherapy. A recent randomized study showed that concurrent administration of 5‐fluorouracil and mitomycin C significantly improved the results of radiotherapy 22. Nevertheless, very few patients with PS of 2 were included in that study. Therefore, both efficacy and tolerability of radiochemotherapy remains unanswered in frail patients. Chemotherapy was used as “neoadjuvant” in 10 of 17 patients who were managed with the combination of these modalities. This strategy has been widely used in bladder preservation protocols 3, 20, but cisplatin‐based chemotherapy has been universally used. In addition, recent data questioned the benefit by “neoadjuvant” chemotherapy in this setting 3. Cisplatin has also been suggested to be superior than carboplatin in radiochemotherapy settings 4. Gemcitabine was used as radiosensitizer in our study. This agent has been popular for unfit patients showing promising results in phase I and II studies 25, 26. Nevertheless, a direct comparison with cisplatin is lacking. More importantly, not all patients received radiotherapy. Current data strongly support the need for definitive local therapy as the standard of care, even in patients who achieve a cT0 after neoadjuvant chemotherapy 27. In concert with recent data 22, our analysis showed that patients treated with combination of radiotherapy and chemotherapy had better oncological outcome than those treated with monotherapy. In fact, only patients treated with the combination had fairly comparable outcome to the other reported series (Table 5).

## Conclusions

Unfit‐for‐surgery patients represent a heterogeneous group. A sizeable fraction is also unfit for the other modalities used in this setting and their therapeutic options are limited. This group of “generally” unfit patients has a poor outcome, receives a variety of therapies, and is underrepresented in clinical trials. In our series, the combination of chemotherapy and radiotherapy seems the most promising option. This population needs to be clearly defined and specifically studied in prospective clinical trials.

## Conflict of Interest

None declared.

## Comorbidities (*n*=45) reported.

**Table:**

0	5 (11)
1	19 (42)
2	13 (29)
3	7 (16)
4	1 (2)
Heart failure	4
Coronary artery disease	11
Hypertension	9
Valve disease	2
Ischemic stroke–Peripheral vascular disease	6
Atrioventricular block	1
Atrial fibrillation	1
Hyperlipidemia	1
Diabetes mellitus	10
Other malignancy	6
Dementia	4
Chronic obstructive airway disease	6
Obesity–sleep apnea	1
renal failure	4
Hypothyroidism	2
Chronic liver disease	1
Connective tissue disease	3
